# The impact of clinical result acquisition and interpretation on task performance during a simulated pediatric cardiac arrest: a multicentre observational study

**DOI:** 10.1007/s43678-022-00313-0

**Published:** 2022-05-19

**Authors:** Carol Rizkalla, Dailys Garcia-Jorda, Adam Cheng, Jonathan P. Duff, Ronald Gottesman, Matthew J. Weiss, Deanna A. Koot, Elaine Gilfoyle

**Affiliations:** 1 Royal College of Surgeons in Ireland, 123 St Stephens Green, Dublin, Ireland; 2 University of Calgary, Calgary, AB Canada; 3 KidSIM Pediatric Simulation Program, Alberta Children’s Hospital, Calgary, AB Canada; 4 Pediatric Emergency Medicine, Alberta Children’s Hospital, Calgary, AB Canada; 5 Pediatrics, Stollery Children’s Hospital, Edmonton, AB Canada; 6 Pediatrics, McGill University, Montreal, QC Canada; 7 Pediatrics, Université Laval Faculté de Médecine, Quebec, QC Canada; 8 Pediatrics, University of Toronto, Toronto, ON Canada

**Keywords:** Patient simulation, Interruptions, Pediatric resuscitation, Team performance, Cardiac arrest, Simulation de patient, Interruptions, Réanimation pédiatrique, Performance d'équipe, Arrêt cardiaque

## Abstract

**Purpose:**

The acquisition and interpretation of clinical results during resuscitations is common; however, this can delay critical clinical tasks, resulting in increased morbidity and mortality. This study aims to determine the impact of clinical result acquisition and interpretation by the team leader on critical task completion during simulated pediatric cardiac arrest before and after team training.

**Methods:**

This is a secondary data analysis of video-recorded simulated resuscitation scenarios conducted during *Teams4Kids* (*T4K*) study (June 2011–January 2015); scenarios included cardiac arrest before and after team training. The scenario included either a scripted paper or a phone call delivery of results concurrently with a clinical transition to pulseless ventricular tachycardia. Descriptive statistics and non-parametric tests were used to compare team performance before and after training.

**Results:**

Performance from 40 teams was analyzed. Although the time taken to initiate CPR and defibrillation varied depending on the type of interruption and whether the scenario was before or after team training, these findings were not significantly associated with the leader's behaviour [Kruskal–Wallis test (*p* > 0.05)]. An exact McNemar’s test determined no statistically significant difference in the proportion of leaders involved or not in interpreting results between and after the training (exact *p* value = 0.096).

**Conclusions:**

Team training was successful in reducing time to perform key clinical tasks. Although team training modified the way leaders behaved toward the results, this behaviour change did not impact the time taken to start CPR or defibrillate. Further understanding the elements that influence time to critical clinical tasks provides guidance in designing future simulated educational activities, subsequently improving clinical team performance and patient outcomes.

**Supplementary Information:**

The online version contains supplementary material available at 10.1007/s43678-022-00313-0.

## Résumé

**But:**

L’acquisition et l’interprétation des résultats cliniques pendant les réanimations sont courantes; toutefois, cela peut retarder les tâches cliniques critiques, ce qui entraîne une augmentation de la morbidité et de la mortalité. Cette étude vise à déterminer l'impact de l'acquisition et de l'interprétation des résultats cliniques par le chef d'équipe sur la réalisation des tâches critiques lors d'un arrêt cardiaque pédiatrique simulé, avant et après la formation de l'équipe.

**Méthodes:**

Il s'agit d'une analyse de données secondaires de scénarios de réanimation simulés enregistrés sur vidéo, réalisés au cours de l'étude *Teams4Kids (T4K)* (juin 2011-janvier 2015); les scénarios comprenaient un arrêt cardiaque avant et après la formation de l'équipe. Le scénario comprenait un document écrit ou un appel téléphonique donnant les résultats en même temps qu’une transition clinique vers la tachycardie ventriculaire sans pouls. Des statistiques descriptives et des tests non paramétriques ont été utilisés pour comparer le rendement de l’équipe avant et après la formation.

**Résultats:**

Les performances de 40 équipes ont été analysées. Bien que le temps nécessaire au déclenchement de la RCP et de la défibrillation ait varié selon le type d'interruption et selon que le scénario se déroulait avant ou après la formation de l'équipe, ces résultats n'étaient pas significativement associés au comportement du leader [test de Kruskal-Wallis (*p* > 0,05)]. Un test exact de McNemar n'a déterminé aucune différence statistiquement significative dans la proportion de dirigeants impliqués ou non dans l'interprétation des résultats entre et après la formation (valeur *p* exacte = 0,096).

**Conclusions:**

La formation en équipe a permis de réduire le temps nécessaire pour effectuer les tâches cliniques clés. Bien que la formation de l'équipe ait modifié le comportement des dirigeants vis-à-vis des résultats, ce changement de comportement n'a pas eu d’incidence sur le temps nécessaire pour commencer la RCP ou la défibrillation. Une meilleure compréhension des éléments qui influencent le temps consacré aux tâches cliniques critiques fournit une orientation pour la conception des futures activités éducatives simulées, améliorant par la suite le rendement des équipes cliniques et les résultats pour les patients.

## Clinician’s capsule

**Table:** 

***What is known about the topic?***
Dealing with delivery of results in a pediatric resuscitation scenario is important as they are common and necessary to patient care.
***What did this study ask?***
What is the impact of results delivery on resuscitation performance, and meeting AHA guidelines for key clinical tasks?
***What did this study find?***
Leader involvement in delivery of results and type of results did not impact time to critical clinical tasks.
***Why does study matter to clinicians?***
Understanding how team training impacts time to performing critical clinical tasks can allow for better tailored educational activities.

## Introduction

The acquisition and interpretation of clinical results is a common and necessary occurrence in pediatric resuscitation settings. To continue effective care for patients, these interruptions (e.g., clinical results delivery via phone call or paper copy) should have minimal impact on factors that determine time to initiate critical clinical tasks such as defibrillation and cardiopulmonary resuscitation (CPR) [1, 2]. Delaying these tasks is much more likely to lead to adverse outcomes, such as increased morbidity and mortality; however, enhancing team/leader performance may mitigate any negative impact delivery of results may have [3]. Thus, understanding how teams deal with these interruptions during simulated cardiac arrest and their effect on completing key clinical tasks (team performance) will provide us with valuable educational strategies.

This study builds upon a previous article that explored the impact of a 1-day team training course on teamwork—it reported time to starting CPR and defibrillation significantly improved after training [4]. However, we do not know whether the leader’s behaviour toward the interruptions impacted the improvement in team performance. This study aims to determine the impact of clinical result acquisition and interpretation by the team leader on critical task completion during simulated pediatric cardiac arrest before and after team training.

## Methods

### Study design, setting, and participants

This is a secondary data analysis of video-recorded simulated resuscitation scenarios conducted during *Teams4Kids (T4K)* study (June 2011–January 2015) [4].

### Scenario

See supplemental data for standardized scenario script (online resource 1). A transition point was scripted to include a planned interruption, which came as a phone call to provide interpretation of the patient's chest XR (CXR)/to report critical labs, or as a paper copy of a CXR/laboratory results to inform the physician of metabolic acidosis, hyperkalemia, hypoglycemia, or hypernatremia. The choice of a phone call or paper results was not random; it was based on the most likely scenario to ensure fidelity and certainty that the interruption would be reasonable based on what, when, and how it was presented (ex. a CXR was delivered if there had not been a CXR already ordered during the scenario and if the team had not looked at a CXR done in the ER). The delivery of results (interruptions) were meant to act as an interfering stimuli to the clinicians’ attention and thought process. Proper interpretation of the results would not lead to a change in immediate patient management; this would have been realized by the teams retrospectively. Results were timed to be introduced seconds before the cardiac arrest, so that the team would be tasked with analyzing the results during the event.

### Variables and outcomes

Median and interquartile range (IQR) were used for quantitative time variables. Categorical variables were represented using frequencies and percentages.

This study's aim is to determine time to completion of critical clinical tasks (initiating defibrillation and CPR) based on type of results (phone call vs paper results) before and after training and leader involvement in results’ interpretation.

### Video review

Video recordings were analyzed for: (a) time to initiating CPR and defibrillation, (b) time taken to analyze results concerning type of results, and (c) behaviour (involved or not involved) of leader concerning type of results. One primary video reviewer analyzed all of videos included in this analysis blinded to the performing team’s institution. Primary reviewer was trained to analyze timed outcomes by a critical care attending who led primary T4K study.

### Data analysis

We summarized participant characteristics and outcomes using descriptive statistics. Non-parametric tests were used, since the variables were not normally distributed. We used median (interquartile range, IQR) for continuous variables and percentage for categorical variables. Changes before and after training were analyzed using Wilcoxon signed-rank test, Kruskal–Wallis, and McNemar test. We used SPSS Statistics for Mac (Version 27.0. Armonk, NY).

## Results

### Participant characteristics

A total of 40 pediatric resuscitation teams participated in 80 video-recorded simulated resuscitation events. Participants’ professional roles, clinical experience, and education are described in supplemental data (Table 1).

**Table 1:** Time taken to analyze results and initiate CPR and defibrillation based on type of results before (PRE) and after (POST) training

Interruption type	Time taken to analyze results in seconds median (IQR)	Time taken to analyze results *N* (percentage of teams)	Time to start CPR in seconds median (IQR)	Time to first shock in seconds median (IQR)
Phone call				
PRE	33 (25.25–45)	0–30 s: 19 (76%) 31–60 s: 4 (16%) Over 60 s: 2 (8%)	49 (19–84.5)	147 (114.5–164)
POST	40 (21–47)	0–30 s: 23 (96%) 31–60 s: 1 (%) Over 60 s: 2 (8%)	24 (20–36)	110 (90–132.75)
Paper results				
PRE	17.5 (14–32.25)	0–30 s: 8 (44%) 31–60 s: 9 (50%) Over 60 s: 1 (6%)	54 (39.25–85)	177 (128.5–260)
POST	13 (7–21)	0–30 s: 5 (31%) 31–60 s: 11 (69%) Over 60 s: –	22 (14–33)	109.5 (79–131.5)

*Time taken to analyze results and initiate CPR and defibrillation* based on type of results delivered.

Results were delivered as paper results in 45/80 scenarios, and phone calls were used in 35/80 scenarios. Table 1 summarizes the time taken to analyze results and time to start CPR and shock based on delivery type (paper or phone).

The time taken to start CPR and defibrillate were not significantly different between teams who received a phone call and teams who received paper results and were not significantly different by leader behaviour [Kruskal–Wallis test (*p* > 0.05)].

### Leader involvement in analyzing results

During the pre-training and post-training scenarios, 32 and 24 leaders, respectively, received new relevant clinical results requiring immediate interpretation. An exact McNemar’s test determined that there was no statistically significant difference in the proportion of leaders involved or not in interpreting results before and after the training (exact *p* value = 0.096). See Table 2 in supplemental data.

The leader behaviour (involvement in interpreting results or not) was significantly related to the type of communication of results (paper or phone call) during the post-training scenarios (Chi-square *p* < 0.001) but not in PRE scenarios (*p* > 0.430). During the post scenarios, most of the leaders did not get involved if they received a phone call (13/17, 76.5%), but when they received paper results, most of them got involved (20/23, 87%).

## Discussion

We found that the teams’ ability to provide timely critical clinical tasks was not related to either the leader's involvement in the acquisition and interpretation of relevant clinical results or the mode of result delivery (paper or phone). Previous work has shown the impact of interruptions on surgical management of patients; however, no studies describe their impact on critical clinical tasks during pediatric resuscitation [1, 3, 5].

### Limitations

As this was a simulation-based study, we could not replicate all unknown variables of a complex clinical environment present in real life. Delivery of results was scripted to happen simultaneously as clinical change, which may not be realistic. Furthermore, since leaders were residents, their leadership experience in resuscitation scenarios was limited. They may experience more stress, and as a result, their performance may suffer more than experienced physicians [1]. Expert attending physicians may be better at simultaneously managing interruptions and maintaining other team functions. As there was no control group without delivery of results, it is difficult to ascertain if they were the cause of delays to CPR/defibrillation provision. There are many other factors that we did not analyze which can impact team performance such as new team members joining the team and noises from other patient rooms (ex. patient alarms), which may also affect team functioning. Improvements pre- and post-training may not have been due to the training itself but may be due to increased comfort with the simulation.

### Clinical implications

Leaders involved themselves more in analyzing paper results after team training. Leaders may have chosen to get involved with paper results as they take less time to analyze than phone, thus maintaining broad situational awareness of resuscitation efforts [4, 6, 7]. However, we did not find leader behaviour influenced whether teams met time to initiating key clinical tasks, and this was likely due to teams’ overall short time taking a phone call and analyzing a paper copy of results (supplemental table 2). The majority of teams took 30 s or less to accomplish these tasks, which would influence whether they met CPR initiation targets of 30 s but not necessarily defibrillation targets of 2 min. Nonetheless, it is well established that the non-technical skills of leadership such as stress management, decision-making, and situational awareness in CPR are crucial to improving team performance; training leaders in these aspects is accessible through simulation courses [6, 8–10].

### Research implications

Future observational work with real-life resuscitation events could describe types of interruptions commonly occurring during resuscitation more accurately. In addition, including attending physicians will allow us to explore the difference prolonged training provides on effectively managing delivery of results and interruptions during resuscitation.

## Conclusion

Team training was successful in reducing time to performing key clinical tasks. Although team training modified the way leaders behaved toward the delivery of results, this change in behaviour did not impact the time taken to start CPR or defibrillate. Further understanding the elements that influence time to critical clinical tasks provides guidance in designing future simulated educational activities which may subsequently improve clinical team performance and patient outcomes.

## Supplementary Information

Below is the link to the electronic supplementary material.

Supplementary file1 (DOCX 45 KB)

## Data Availability

Not applicable.
