# Supplementary material for: The impact of clinical result acquisition and interpretation on task performance during a simulated pediatric cardiac arrest: a multicentre observational study
Source: CJEM. 2022 May 19;24(5):529–34. doi: 10.1007/s43678-022-00313-0 (PMC9345827; doi:10.1007/s43678-022-00313-0)
Supplement: Supplementary file 1 — Supplementary file1 (DOCX 45 KB) [file 43678_2022_313_MOESM1_ESM.docx]

Article title: Impact of delivery of results on clinical care during simulated pediatric cardiac arrest: A multicenter observational study

Journal name: Canadian journal of emergency medicine

Author list:

Carol Rizkalla^1^

Dailys Garcia Jorda, PhD^2^

Adam Cheng, MD^4^

Jonathan P Duff, MD^5^

Ronald Gottesman, MD^6^

Matthew J. Weiss, MD^7^

Deanna Koot, RN^3^

Elaine Gilfoyle, MD^8^

Author Affiliation:

^1^ Royal College of Surgeons in Ireland, Dublin, Ireland, [carolrizkalla@rcsi.com](mailto:carolrizkalla@rcsi.com)

^2^ University of Calgary, Calgary, Alberta, [dailys.garciajorda@ucalgary.ca](mailto:dailys.garciajorda@ucalgary.ca)

^3^ Alberta Children's Hospital, KidSIM Pediatric Simulation Program, Calgary, Alberta, [dkoot@me.com](mailto:dkoot@me.com)

^4^ Alberta Children's Hospital, Pediatric Emergency Medicine, Calgary, Alberta, [chenger@me.com](mailto:chenger@me.com)

^5^ Stollery Children’s Hospital, Pediatrics, Edmonton, Alberta, [jduff@ualberta.ca](mailto:jduff@ualberta.ca)

^6^ McGill University, Pediatrics, Montreal, Quebec, [ronald.gottesman@mcgill.ca](mailto:ronald.gottesman@mcgill.ca)

^7^ Université Laval Faculté de Médecine, Pediatrics, Quebec City, Quebec, [Matthew-John.Weiss@mail.chudequebec.ca](mailto:Matthew-John.Weiss@mail.chudequebec.ca)­

^8^ University of Toronto, Pediatrics, Toronto, Ontario, [elaine.gilfoyle@sickkids.ca](mailto:elaine.gilfoyle@sickkids.ca)

Corresponding author: Carol Rizkalla, 104 Panatella Rise NW, 353-83-891-8473, carolrizkalla@rcsi.com

**Online-Only Supplements**

**Table 1** Professional and educational characteristics of study participants

| **Characteristics of Study Participants** | **N (%) of Cohort^a^ (n=255)** |
| --- | --- |
| **Study Site** |  |
| A | **104 (40.8)** |
| B | **45 (17.6)** |
| C | **49 (19.2)** |
| D | **57 (22.4)** |
| **Professional role** |  |
| Resident physician | **87 (34.1)** |
| 0-1 yr/PGY1 | **31 (12.4)** |
| 1-2 yr/PGY2 | **49 (19.6)** |
| 2-3 yr/PGY3 | **43 (17.3)** |
| 3-4 yr/PGY4 | **18 (7.2)** |
| 4-5 yr/PGY5 | **21 (8.4)** |
|  |  |
| ICU nurse practitioner | **2 (0.8)** |
| Registered nurse | **110 (43.1)** |
| Registered respiratory therapist | **56 (22.0)** |
|  |  |
| **Years of experience (other than residents)** |  |
| 5- 10 yr | **46 (18.5)** |
| 11-15 yr | **25 (10.0)** |
| 16-20 yr | **7 (2.8)** |
| >20 yr | **9 (3.6)** |

^a^Years of experience refers to the number of years worked in current role.

^b^ n=249 for years of experience due to data loss from 1 team.

**Table 2** Leader *involvement in analysing results*

| **Leader behavior** | **Pre team-training** | **Post team-training** |
| --- | --- | --- |
| **Involved** | 32 | 24 |
| **Not involved** | 8 | 16 |

**Simulation Scenario Shock Version A**

**Overall Case Progression:** Babe admitted with vomiting and fever for rehydration. IV access lost. Initially responds to fluid bolus but deteriorates again, fluid doesn’t work and makes patient desat further. Turns out to be cardiogenic shock/myocarditis. Will require intubation. V-Tach Arrest. Will have some critical labs that will need to be addressed (severe metabolic acidosis, hyperkalemia, hypoglycemia, hypernatremia) - these lab results are offered during critical time in scenario.

**Location (Scenario Scene):** inpatient/ward in a patient room

Initial Set-Up:

- Mannequin: Laerdal-SimBaby
- Monitors: none initially
- Hemodynamic/monitoring: none
- Physical props/equipment: patient will have IV in situ with clamped drainage bag.
- Respiratory: patient will have nasal prongs on at 2 LPM on a high-flow (15 LPM+) oxygen meter
- Make up /moulage: infant dressed in hospital sleeper. Diaper on is dry.
- Multi media:
  - Patient Chart including:
    - ED RN record, ED MD record, history & physical exam from peds resident and co-signed by paediatrician/hospitalist, orders include clear fluid diet/Breastfeed ad lib, rehydration & Tylenol
  - From ER: CBG (metabolic acidosis), CBC & lytes (low CO2, BUN high, creat (slightly high), glucose 2.1), CXR (non-intubated, consolidation to one side), abdominal ultrasound report (couldn’t visualize appendix, abnormal gas pattern, no perforation)
  - Transition #2: CBG
  - Transition #5: CXR (intubated, progressive pulmonary edema, cardiomegaly

Initial Mannequin/Computer Set Up:

- HR 195 with increasing trend
- RR 60 – no crackles to start, Increased WOB
- BP 60/28 with falling trend
- O2 sat 98% on 2 LPM nasal prongs

**Instructor Roles:**

Scenario Coordinator/Mannequin/Computer Operations

(1) Mannequin/Computer Operations - this instructor will also provide assessment data to the participants.

(2) (OPTIONAL) If the computer operator does not normally interact with the participants at your site or room set up does not allow for this then another instructor will be assigned to coordinate the scenario including providing assessment data to the participants.

Confederates

(3) Confederate - ward RN to provide history, distractions, and prompts as timed in the script

**Scenario**

**Case Introduction:** (Instructor reads this script to the participants that are waiting outside the simulation room immediately before the team begins scenario) “You are part of the hospital code blue team and are called to a code on inpatient unit. The crash cart and airway equipment were already brought into the room for you.”

** Participants then enter the room **

This history to be given by the ward RN (the RN that called the code) if requested by the team:

“Billy (patient) is a 8 month old male who was admitted for vomiting, fever and fluid rehydration through ED came to unit at 2200h last night. Mom went home for a few minutes to shower & change. While doing assessment found the patient to be tachycardic, hypotensive and difficult to rouse so I called a code”

**Available Collateral History: (**information given if requested by the ward RN)

- Weight 9 Kg
- Admitted last night with 3 days of vomiting, and fever
- Received 2 x 20 cc/kg fluid boluses in ED but continued to vomit so was admitted for rehydration. Received 1 more 20 cc/kg bolus over night. Remained awake, alert & appropriate since admission, until now
- ED Record: abdominal ultrasound-couldn’t visualize appendix, abnormal gas pattern, no perforation. CBC showing elevated WBC, Left shift, lytes showing hypokalemia, hypochloremia, slight metabolic acidosis (low CO2)

| **Scenario Transitions/ Evolution** | **Effective Management** | **Ineffective Management & Consequences of Ineffective Management** | **Notes** |
| --- | --- | --- | --- |
| **1. Assessment & recognition of poor perfusion/shock state**  Time: 0 minutes   - HR 195 with increasing trend - BP: 60/28 with falling trend - O2: SaO2 98% on 2L nasal prongs - RR: 60 shallow, indrawing - Chest: clear - CNS: eyes closed, no cry | Systematic assessment and intervention to support ABCD’s:   - Position airway - Provide adequate supplemental oxygen (non-re-breather) - Pulse check and CVS assessment - Places on monitors, BP check - Prioritize IV/IO access - Assesses CNS/LOC | - Do not provide adequate supplemental oxygen - No rapid pulse check - Forgets to place on monitors or check BP - Access not assessed or addressed/initiated   *********  Status remains unchanged  **If management is ineffective** at 3 minutes:  Confederate ward RN or consult service if help has been called for can interject:   - Offer to continue/attempt PIV if it has not been delegated or attempted - Suggest placing the babe on the monitor - Report that last record BP on the unit was 86/48 repeated x 3   At 6 minutes if management is ineffective:   - Skip to transition #4: deteriorates | Patient weight available from ward RN – 9kg  Info available if asked from computer/instructor:   - CNS: lethargic, eyes closed, no crying, only occasional moans, no resistance to starting IV, temp 39 axilla - CVS: cap refill 5 seconds, pale and mottled extremities, no cyanosis - Resp: indrawing (as on mannequin, nasal flaring, no obvious head bobbing, no upper airway noise - Integ: skin intact, no rash, multiple poke sites to extremities   Changes required during scenario:  Computer/mannequin:  ** Adjust SaO2 based on oxygen delivery device and flow-rate.  ACCESS - must go through process of obtaining access. Note - the team gets IV on first attempt and the instructor acting as Ward RN must set up drainage bag |

| **Scenario Transitions/ Evolution** | **Effective Management** | **Ineffective Management & Consequences of Ineffective Management** | **Notes** |
| --- | --- | --- | --- |
| **2. Recognition of need to give rapid fluid bolus & check glucose**   - HR: no change - BP: no change - O2: SaO2 response based on supplemental oxygen device and flow-rate - RR: no change | - Orders 20 cc/kg fluid bolus, either push or on pressure bag - Check chemstrip | - Orders 20 cc/kg bolus, which gets put on pump - Doesn’t check chemstrip   *****  No suggestion or intervention to prompt further intervention.  At 6 minutes if management is ineffective:  Skip to transition #4: deteriorates | Info available if asked from computer operator/instructor (no changes from previous):   - Chemstrip: ward RN offers to collect and/or run the sample if ordered (after 15 seconds reports result, 2.8) - CBG (must be collected): results take 4 minutes and then are provided by ward RN directly to team leader |
| **3. Resolving shock if fluid bolus administered effectively**  Time: immediately following bolus administration   - HR: decrease by 10 over 3 minutes - BP: systolic increases by 15 over 3 minutes - O2: sat 92% on non-re-breather of by BVM device - RR: 60, indrawing unchanged - Chest: unchanged - CNS: eyes closed but moans to deep pain | - Cap refill, HR, BP, LOC recheck - Recognizes improvement in perfusion - Orders labs (someone on team should poke, ward RN – confederate – offers to transfer to tubes and send to lab) | - Doesn’t reassess after fluid bolus - Doesn’t follow up on chemstrip - Calls lab to come poke | - Have to tell them cap refill is 4 sec - CNS: unchanged |

| **Scenario Transitions/ Evolution** | **Effective Management** | **Ineffective Management & Consequences of Ineffective Management** | **Notes** |
| --- | --- | --- | --- |
| **4. Deteriorates**   - HR: 200 with increasing trend - BP: 55/30 - O2: SaO2 92% and falling - RR: 60 - Chest: crackles bilaterally - CNS: unresponsive, eyes closed, moans to deep pain | - Recognize deterioration - Repeat fluid bolus 20 cc/kg either push or pressure bag | - Fluid bolus on pump | Have to tell them cap refill 5 seconds, still unresponsive (moans with painful stim, doesn’t open eyes).   - CNS: unresponsive, eyes closed, moans to pain but no eye opening - CVS: cap refill 5-6 seconds, pale and mottled, no cyanosis - Resp: indrawing (as on mannequin), nasal flaring, no obvious head bobbing, no upper airway noise - Integ: skin intact, no rash, multiple poke sites to extremities |
| **5. Decreasing O2 saturations & becomes apneic**   - HR: no change - BP: no change - O2: SaO2 falling - RR: falling – apneic | - Recognize lack of improvement and decreasing sats - Orders CXR or asks to see CXR done in ED - Respiratory failure | - Doesn’t recognize lack of improvement - Stats get lower and lower - Doesn’t order CXR   Progress to transition #7 at 10 minutes | - Apnea - O2 sats 90% and falling, RR 0, HR same, BP same |
| **6. Recognizes respiratory failure & cardiogenic shock** | - Call for help (ICU or cardiology) - Orders echo - Orders inotrope infusion (dobutamine? dopamine?) - Recognizes need to intubate and prepares (airway equipment, assign person to intubate, intubation meds?) - Recognized potential for deterioration during intubation (epi dose ready?) | - Doesn’t recognize change in status - Doesn’t consider items listed to the left |  |

| **Scenario Transitions/ Evolution** | **Effective Management** | **Ineffective Management & Consequences of Ineffective Management** | **Notes** |
| --- | --- | --- | --- |
| **7. V-tach arrest (timed)**  Time: 10 minutes   - HR: 200, V-tach, lose pulse - BP: undetectable - O2: sat undetectable - RR: apneic | - Recognizes change in rhythm and loss of pulse - Chest compression initiated immediately - Defibrillation - Give dose of epi | - Doesn’t recognize deterioration - Doesn’t start chest compressions - Doesn’t defibrillate or does it incorrectly (wrong electricity dose or synchronizes inappropriately) - Doesn’t give epi - Wrong dose epi | OPTIONS for distractor (does this at exact time of change in rhythm):  (1) Ward RN can call up CXR when intubating and says “you should look at this right away”:   - If the team has not looked at CXR done in ER during the scenario - If there has not been a CXR ordered during the scenario as of yet   (2) Ward RN report critical labs and hand to leader and say, “There are some critical values. You should look at these right away.” |
| **8: Recovers HR & pulse (30 seconds to 1 minute after epi)**   - HR: increase to 140 (NSR), pulse weak - BP: detectable x 1 reading, if repeats or cycles BP then 50/20 - O2: SaO2 undetectable initially then recovers, reading slowly increase to 907% over 30 seconds - RR: bagging | - Calls again for help - Checks on status on inotrope infusion - Calls for status on getting echo - Call ICU for transfer | - Doesn’t ask for help - Doesn’t consider that patient needs to be transferred to ICU | SCENARIO COMPLETE:   - TIME: 15 MINUTES |

**Simulation Scenario Shock Version B**

**Overall Case Progression:** Babe admitted with bronchiolitis. IV access lost and no fluids administered. Initially responds to fluid bolus but deteriorates again with evolving shock but worsening respiratory distress with further fluid challenge. Will require intubation. Turns out to be cardiogenic shock/myocarditis. V-Tach Arrest. Will have distractors including radiologist wanting to offer interpretation of CXR done in ER.

**Location (Scenario scene):** inpatient/ward in a patient room

Initial Set-Up:

- Mannequin: Laerdal SimBaby
- Monitors: none initially
- Hemodynamic/Monitoring: none
- Physical props/equipment: patient will have IV in situ with clamped drainage bag.
- Respiratory: patient will have nasal prongs on at 2 LPM on a high-flow (15 LPM+) oxygen meter
- Make up /moulage: infant dressed in hospital sleeper. Diaper on is dry.
- Multi media:
  - Patient Chart including
    - ED RN record, ED MD record, history & physical exam from peds resident and co-signed by paediatrician/hospitalist, orders include clear fluid diet/Breastfeed ad lib, rehydration & Tylenol
  - From ER: CBG (metabolic acidosis), CBC & lytes (low CO2, BUN high, creat (slightly high), glucose 2.1), CXR (non-intubated, bilateral increased pulmonary vascular markings consistent with pulmonary edema)
  - Transition #2: CBG
  - Transition #5: CXR (intubated, progressive pulmonary edema, cardiomegaly)

Initial Mannequin/Computer Set Up:

- HR 195 with increasing trend
- RR 60 – decreased A/E to bases, crackles unilaterally, Increased WOB
- BP 60/28 with falling trend
- O2 sat 98% on 2 LPM via NP (started by RN or resident when assessing babe and calling code team)

**Instructor Roles:**

Scenario Coordinator/Mannequin/Computer Operations

(1) Mannequin/Computer Operations - this instructor will also provide assessment data to the participants.

(2) (OPTIONAL) If the computer operator does not normally interact with the participants at your site or room set up does not allow for this then another instructor will be assigned to coordinate the scenario including providing assessment data to the participants.

Confederates

(3) Confederate - ward RN to provide history, distractions, and prompts as timed in the script

(4) Confederate - radiologist calling in transition #7 to offer interpretation of initial chest x-ray (may to be scenario coordinator/computer operations depending on number of instructors available

**Scenario**

**Case Introduction:** (Instructor reads this script to the participants that are waiting outside the simulation room immediately before the team begins scenario) “You are part of the hospital code blue team and are called to a code on inpatient unit. The crash cart and airway equipment were already brought into the room for you.”

** Participants then enter the room **

This history to be given by the ward RN (the RN that called the code) if requested by the team:

“Joe (patient) is a 10 month old male who was admitted for increased WOB - diagnosis: bronchiolitis - through ED came to unit 3 hours ago. I came in to assess him and start a PIV (that one is not flushing) and oxygen stats 91% on 1 LPM by nasal prongs increased to 2 LPM with little response, and his work of breathing is as you see now. Vital signs HR 160, BP 85/42. I found him difficult to arose so I called a code.”

**Available Collateral History:** (information given if requested by the ward RN)

- Weight 10 Kg
- Admitted through ER to units 3 hours ago
- 3 day history of runny nose, increased WOB, febrile x 5 days. Second visit to ER and told likely “has a virus.”
- Has not taken solid food in 2 days and nuzzles at breast for comfort but mom thinks that intake is poor. Small wet diaper x 1 last night in ER. Current diaper is dry as on assessment.
- Had CXR and bloodwork done.
- Bloodwork completed in ER. Lab results available in chart/electronic health record.
- PIV interstitial when arrived on unit unable to infuse therefore no fluids given x 3+hours. Colleague had just started that PIV but it does not flush either. Was about to d/c it and make another attempt.

| **Scenario Transitions/ Evolution** | **Effective Management** | **Ineffective Management & Consequences of Ineffective Management** | **Notes** |
| --- | --- | --- | --- |
| **1. Assessment & recognition of poor perfusion/shock state**  Time: 0 minutes   - HR 195 with increasing trend - BP: 60/28 with falling trend - O2: SaO2 98% on 2L nasal prongs - RR: 60 shallow, indrawing - Chest: clear - CNS: eyes closed, no cry | Systematic assessment and intervention to support ABCD’s:   - Position airway - Provide adequate supplemental oxygen (non-re-breather) - Pulse check and CVS assessment - Places on monitors, BP check - Prioritize IV/IO access - Assesses CNS/LOC | - Do not provide adequate supplemental oxygen - No rapid pulse check - Forgets to place on monitors or check BP - Access not assessed or addressed/initiated   *********  Status remains unchanged  **If management is ineffective** at 3 minutes:  Confederate ward RN or consult service if help has been called for can interject:   - Offer to continue/attempt PIV if it has not been delegated or attempted - Suggest placing the babe on the monitor - Report that last record BP on the unit was 86/48 repeated x 3   At 6 minutes if management is ineffective:   - Skip to transition #4: deteriorates | Patient weight available from ward RN – 10kg  Info available if asked from computer/instructor:   - CNS: lethargic, eyes closed, no crying, only occasional moans, no resistance to starting IV, temp 39 axilla - CVS: cap refill 5 seconds, pale and mottled extremities, no cyanosis - Resp: indrawing (as on mannequin, nasal flaring, no obvious head bobbing, no upper airway noise - Integ: skin intact, no rash, multiple poke sites to extremities   Changes required during scenario:  Computer/mannequin:  ** Adjust SaO2 based on oxygen delivery device and flow-rate.  ACCESS - must go through process of obtaining access. Note - the team gets IV on first attempt and the instructor acting as Ward RN must set up drainage bag. |

| **Scenario Transitions/ Evolution** | **Effective Management** | **Ineffective Management & Consequences of Ineffective Management** | **Notes** |
| --- | --- | --- | --- |
| **2. Recognition of need to give rapid fluid bolus & check glucose**   - HR: no change - BP: no change - O2: SaO2 response based on supplemental oxygen device and flow-rate - RR: no change | - Orders 20 cc/kg fluid bolus, either push or on pressure bag - Check chemstrip | - Orders 20 cc/kg bolus, which gets put on pump - Doesn’t check chemstrip   *****  No suggestion or intervention to prompt further intervention.  At 6 minutes if management is ineffective:   - Skip to transition #4: deteriorates | Info available if asked from computer operator/instructor (no changes from previous):   - Chemstrip: ward RN offers to collect and/or run the sample if ordered (after 15 seconds reports result, 2.8) - CBG (must be collected): results take 4 minutes and then are provided by ward RN directly to team leader |
| **3. Resolving shock if fluid bolus administered effectively**  Time: immediately following bolus administration   - HR: decrease by 10 over 3 minutes - BP: systolic increases by 15 over 3 minutes - O2: sat 92% on non-re-breather of by BVM device - RR: 60, indrawing unchanged - Chest: unchanged - CNS: eyes closed but moans to deep pain | - Cap refill, HR, BP, LOC recheck - Recognizes improvement in perfusion - Orders labs (someone on team should poke, ward RN – confederate – offers to transfer to tubes and send to lab) | - Doesn’t reassess after fluid bolus - Doesn’t follow up on chemstrip - Calls lab to come poke | - Have to tell them cap refill is 4 sec - CNS: unchanged |

| **Scenario Transitions/ Evolution** | **Effective Management** | **Ineffective Management & Consequences of Ineffective Management** | **Notes** |
| --- | --- | --- | --- |
| **4. Deteriorates**   - HR: 200 with increasing trend - BP: 55/30 - O2: SaO2 92% and falling - RR: 60 - Chest: crackles bilaterally - CNS: unresponsive, eyes closed, moans to deep pain | - Recognize deterioration - Repeat fluid bolus 20 cc/kg either push or pressure bag | - Fluid bolus on pump | Have to tell them cap refill 5 seconds, still unresponsive (moans with painful stim, doesn’t open eyes).   - CNS: unresponsive, eyes closed, moans to pain but no eye opening - CVS: cap refill 5-6 seconds, pale and mottled, no cyanosis - Resp: indrawing (as on mannequin), nasal flaring, no obvious head bobbing, no upper airway noise - Integ: skin intact, no rash, multiple poke sites to extremities |
| **5. Decreasing O2 saturations & becomes apneic**   - HR: no change - BP: no change - O2: SaO2 falling - RR: falling – apneic | - Recognize lack of improvement and decreasing sats - Orders CXR or asks to see CXR done in ED - Respiratory failure | - Doesn’t recognize lack of improvement - Stats get lower and lower - Doesn’t order CXR   Progress to transition #7 at 10 minutes | - Apnea - O2 sats 90% and falling, RR 0, HR same, BP same |
| **6. Recognizes respiratory failure & cardiogenic shock** | - Call for help (ICU or cardiology) - Orders echo - Orders inotrope infusion (dobutamine? dopamine?) - Recognizes need to intubate and prepares (airway equipment, assign person to intubate, intubation meds?) - Recognized potential for deterioration during intubation (epi dose ready?) | - Doesn’t recognize change in status - Doesn’t consider items listed to the left |  |

| **Scenario Transitions/ Evolution** | **Effective Management** | **Ineffective Management & Consequences of Ineffective Management** | **Notes** |
| --- | --- | --- | --- |
| **7. V-tach arrest (timed)**  Time: 10 minutes   - HR: 200, V-tach, lose pulse - BP: undetectable - O2: sat undetectable - RR: apneic | - Recognizes change in rhythm and loss of pulse - Chest compression initiated immediately - Defibrillation - Give dose of epi | - Doesn’t recognize deterioration - Doesn’t start chest compressions - Doesn’t defibrillate or does it incorrectly (wrong electricity dose or synchronizes inappropriately) - Doesn’t give epi - Wrong dose epi | OPTIONS for distractor (does this at exact time of change in rhythm):  (1) Radiologist calls insisting to talk to MD about interpretation of CXR that was done in ER:   - If the team has not looked at CXR done in ER during the scenario - If there has not been a CXR ordered during the scenario as of yet   (2) Lab tech is on the phone to report critical labs and must speak with doctor |
| **8: Recovers HR & pulse (30 seconds to 1 minute after epi)**   - HR: increase to 140 (NSR), pulse weak - BP: detectable x 1 reading, if repeats or cycles BP then 50/20 - O2: SaO2 undetectable initially then recovers, reading slowly increase to 907% over 30 seconds - RR: bagging | - Calls again for help - Checks on status on inotrope infusion - Calls for status on getting echo - Call ICU for transfer | - Doesn’t ask for help - Doesn’t consider that patient needs to be transferred to ICU | SCENARIO COMPLETE:   - TIME: 15 MINUTES |
